# Relationship between sedentary behaviour and anxiety symptoms among youth in 24 low- and middle-income countries

**DOI:** 10.1371/journal.pone.0241303

**Published:** 2020-10-26

**Authors:** Ming-Hui Wang, Dian-Min Xiao, Ming-Wei Liu, Yuan-An Lu, Qi-Qiang He

**Affiliations:** 1 School of Health Sciences, Wuhan University, Wuhan, China; 2 Gannan Medical University, Ganzhou, China; 3 Environmental Health Laboratory, Department of Public Health Sciences, University Hawaii at Manoa, Honolulu, HI, United States of America; Iwate Medical University, JAPAN

## Abstract

**Background:**

Anxiety is burdensome and common in youth. Sedentary behaviour has been identified as potentially modifiable dangerous factors for many diseases. Nevertheless, little is known about the relationship between sedentary behaviour and the risk of anxiety symptoms in youth. Therefore, we aimed to examine the association among youth in 24 low- and middle-income countries (LMICs).

**Methods:**

Data from the Global School-based Student Health Survey (GSHS) were analyzed in 59587 youth aged 12–15 years. Most of the country-wide data were nationally representative. Anxiety symptoms were self-reported. Multivariable logistic regression and meta-analyses of country-wise estimates were conducted.

**Results:**

The prevalence of anxiety symptoms was 10.3%. Countrywide meta-analysis demonstrated that sedentary behaviour of >2 h/day (vs.≤2 h/day) was associated with an increased risk of anxiety symptoms (OR = 1.22; 95% CI = 1.10–1.37).

**Conclusions:**

This study provides multi-national evidence of the dangerous effect of sedentary behaviour against anxiety symptoms among youth in LMICs. Decreasing the level of sedentary behaviour during adolescence could be an important target for reducing the prevalence of anxiety.

## 1. Introduction

Mental health disorders are the leading cause of disability all over the world [1]. World Health Organization (WHO) declared that more than four-fifth of people suffered from mental disorders in the low- and middle-income countries (LMICs). Anxiety is among the main symptoms of mental disorders. Anxiety symptoms refer to a loss of interest in previously enjoyed activities, tiredness, restlessness, difficulty controlling worry and other symptoms [2]. Anxiety symptoms decrease the likeliness of finishing school, finding a job and enjoying a high quality of life [3]. It estimates that about 6.5% of youth under 18 years meet diagnostic criteria for anxiety symptoms [4]. Considering that mental disorders may have originated from youths, it is incredibly significant to know the correlatives of mental health to recognize potentially modifiable risk factors [5]. Therefore, it is urgent to take some measures to prevent this type of mental disorder.

Sedentary behaviour has become a significant component of our daily lives [6]. It includes any conscious behaviour characterized by an energy expenditure ≤1.5 metabolic equivalents, while in a reclining, lying or sitting posture [7]. Youth tend to spend more time on sedentary behaviour. For instance, a recent study indicated that over one-third of 72,845 kids from 34 diverse countries took at least 3 hours in sedentary behaviour every day [8]. Recent studies found that avoiding sedentary behaviour can alleviate or prevent anxiety symptoms in youth [9]. Nevertheless, the evidence is complex. One study found no association between sedentary activity and anxiety symptoms in youngsters [10]. Besides, only a few studies have explored the association between sedentary activity and anxiety symptoms among youth in LMICs [11, 12]. Furthermore, most prior studies on the relationship between sedentary behaviour and anxiety symptoms are often conducted in a single country, and the sample size is small [11]. Therefore, we performed this multinational study to clarify the association of sedentary behaviour with anxiety symptoms among youth living in 24 LMICs.

## 2. Methods

### 2.1. Study population

Data was openly available from the Global school-based Student Health Survey (GSHS). Details about this investigation were available at http://www.cdc.gov/gshs and http://www.who.int/chp/gshs.. GSHS was a coefficient supervision project conducted by World Health Organization and the United States Centers for Disease Control and Prevention. The main aim of this survey was to explore risk and protective factors of non-communicable sickness. In short, the survey conducted a standardized two-stage cluster sampling process to select representative country samples. First of all, schools were selected with probability proportional to sample size. Then classes within schools were randomly selected. Everyone in sampled classes can take part. The questionnaire was translated into local languages. Students recorded their response on computer scanable sheets. All surveys were approved in each country by both an institutional review board or ethics committee and a national government administration.

**Fig 1:**
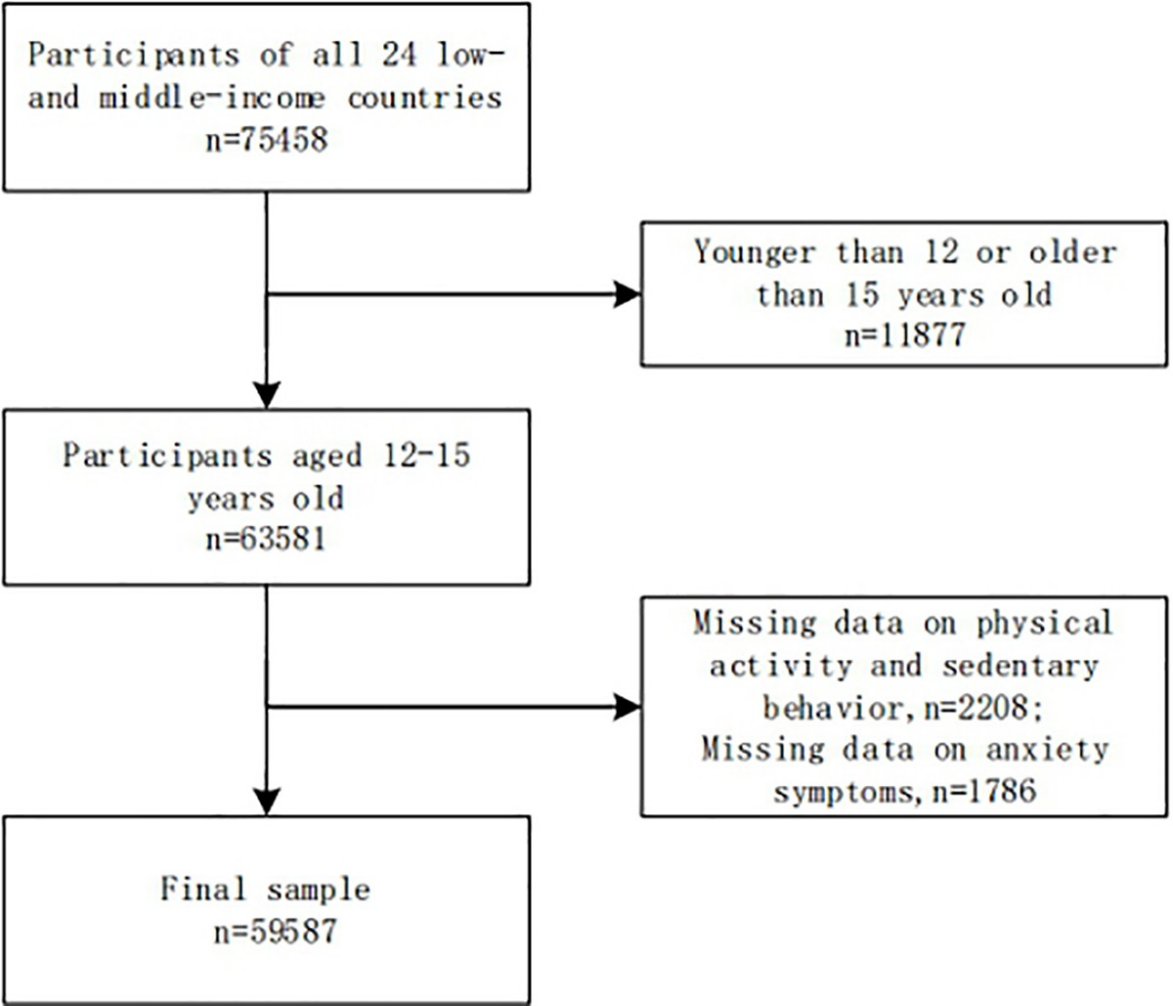
Flow chart of the study sample.

We included all the datasets that comprised variables relation to our analyses. The question about anxiety symptoms was only available in the questionnaire for investigations conducted during year 2003 and year 2008. We selected the most recent one if there were more than two datasets from one country during this period. A total of 24 countries were included in this study. All countries were LMICs based on the World Bank classification at the period of the study. Data were nationally representative for 20 countries except for 4 countries where the study was only conducted in selected areas: Tanzania (Dar Es Salaam), China (Beijing), Ecuador (Quito), and Venezuela (Lara). The analysis was restricted to participants aged 12–15 years since most of them were within this age range. Fig 1 depicts a detailed description of the inclusion and exclusion criteria applied in this study.

### 2.2. Anxiety symptoms

Participants who answered “most of the time”, or “always” to the question “During the past 12 months, how often have you been so worried about something that you could not sleep at night?” were defined to have anxiety symptoms [3].

### 2.3. Sedentary behaviour

Sedentary behaviour was assessed with the question “How much time do you spend during a typical or usual day sitting and watching television, playing computer games, talking with friends, or doing other sitting activities?” This question has six answer options: < 1, 1–2, 3–4, 5–6, 7–8, and ≥8 h/day. This variable was used as a dichotomized variable (>2h/day or not), in accordance with previous research [6].

### 2.4. Covariates

Age, sex, country, smoking status, physical activity, alcohol intake, fruit and vegetable intake, whether one had been bullied were used as control variables in the study. Physical activity was defined by this question: “During a typical or usual week, on how many days are you physically active for a total of at least 60 minutes per day?” It excludes physical education or gym classes. In accordance with previous research [8], the numbers of physically active days (≥60 min/d) were categorized into <5 days/wk and ≥5 days/wk. Given that only the frequency of fruit and vegetable intake was available in the GSHS data, we used ≥5 times/d as a proxy cut-off to measure adequate fruit and vegetable intake. Smoking status, alcohol intake and being bullied was assess by three isolated questions separately: “During the past 30 days, did you smoke cigarettes?”; “During the past 30 days, did you have at least one drink containing alcohol?” and “During the past 30 days, on how many days were you bullied?”; These three variables were dichotomized into having “ever” or “not”.

### 2.5. Statistical analyses

Multivariable logistic regression analysis was conducted to explore the association of sedentary behaviour with anxiety symptoms. The regression analyses were adjusted for sex, age, physical activity, being bullied, fruit and vegetable intake, smoking status and alcohol intake where applicable (Not all countries had all measurements, see tables for details). Pooled estimates were procured by combining the estimates of each country with random-effect meta-analyses, including the prevalence of anxiety symptoms, and odds ratios (OR) of anxiety symptoms according to sedentary behaviour. The Higgin's I^2^ statistic was calculated to assess the level of between-country heterogeneity. We conducted a conventional approach of a weighted method for pooled OR. Subgroup analyses were performed ground on region and sex. Taylor linearization methods were used in all analyses to account for the sophisticated sampling design. Specifically speaking, SAS survey procedures were used to apply relevant weighting variables provided in the GSHS datasets. We used Stata 14.1 (Stata Corp LP, College station, Texas) to obtain pooled estimates. All other analyses were performed using SAS (version 9.4; SAS Institute Inc, Cary, NC). We considered differences as significant at p < 0.05.

## 3. Results

Table 1 summarizes descriptive characteristics of the study population. The final sample consisted of 59587 youth aged 12–15 years with a mean age of 13.8 years old and 47% were boys, the pooled estimates of overall prevalence rates was 10.3% (95% CI = 8.6,12.0) for anxiety symptoms. Sedentary behaviour of >2 h/day was observed in 32.7% of the youth (ranging from 9.6% in Myanmar to 53.5% in Saint Lucia). The region-based pooled prevalence of high sedentary time was the highest in Americas region (39.0%) and the lowest in East and Southeast Asia region (26.4%). The prevalence of anxiety symptoms was lower in Americas and East and Southeast Asian regions compared to African and Eastern Mediterranean regions.

**Table 1:** Basic characteristic of the study sample^a^.

Region/Country	year	Sample N	Weighted N	Age (years)	Boys (%)	Sedentary behavior>2h/d (%)	Anxiety symptoms (%)
Africa		9288	1544949	13.9	45.9	34.8(28.8,40.8)	13.1(7.4,18.8)
Botswana	2005	1307	72316	14.3	45.6	34.5(31.9,37.1)	18.0(15.9,20.1)
Kenya	2003	2434	931282	13.9	46.6	36.5(34.6,38.4)	13.6(12.2,15.0)
Seychelles	2007	1038	5317	13.6	49.1	50.8(47.8,53.8)	11.5(9.6,13.4)
Uganda	2003	1779	259138	14.3	47.1	27.0(24.9,29.1)	9.2(7.9,10.5)
United Republic of Tanzania	2006	1675	97099	13.0	46.1	28.8(26.6,31.0)	3.0(2.2.,3.8)
Zambia	2004	1055	179797	13.9	50.3	31.4(28.6,34.2)	23.6(21.0,26.2)
Americas		13797	1511814	13.7	44.2	39.0(31.4,46.6)	9.7(6.7,12.6)
Argentina	2007	1464	1221663	14.1	45.6	48.6(46.0,51.2)	10.8(9.2,12.4)
Ecuador	2007	4256	132931	13.4	48.4	28.7(27.3,30.1)	7.8(7.0,8.6)
Grenada	2008	1172	5543	13.7	42.1	41.3(38.5,44.1)	10.0(8.3,11.7)
Guyana	2004	1001	33023	14.1	46.3	37.0(34.0,40.0)	12.8(10.7,14.9)
Saint Lucia	2007	1015	8278	13.7	44.4	53.5(50.4,56.6)	10.5(8.6,12.4)
Saint Vincent and Grenadines	2007	1044	5760	13.5	45.8	38.7(35.7,41.7)	12.6(10.6,14.6)
Venezuela	2003	3845	104616	13.2	47.2	25.4(24.0,26.8)	3.6(3.0,4.2)
Eastern Mediterranean		11238	4057294	13.9	50.2	29.0(23.5,34.5)	14.4(9.8,19.0)
Djibouti	2007	917	7348	14.3	59.5	31.5(28.5,34.5)	14.0(11.8,16.2)
Egypt	2006	4536	2474828	13.2	51.5	22.0(20.8,23.2)	8.3(7.5,9.1)
Jordan	2007	1497	203352	14.4	45.6	38.0(35.5,40.5)	17.8(15.9,19.7)
Morocco	2006	1871	891730	14.0	52.1	29.8(27.7,31.9)	13.1(11.6,14.6)
Tunisia	2008	2417	480036	13.6	49.4	24.1(22.4,25.8)	18.9(17.3,20.5)
East and Southeast Asia		25264	9851639	13.7	48.2	26.4(19.1,33.7)	5.2(3.4,7.0)
India	2007	6892	1626314	13.9	57.5	23.0(22.0,24.0)	7.5(6.9,8.1)
Indonesia	2007	2951	2968563	13.8	49.3	34.0(32.3,35.7)	7.4(6.5,8.3)
Myanmar	2007	2207	1228969	13.6	49.5	9.6(8.4,10.8)	1.8(1.2,2.4)
Sri Lanka	2008	2425	882556	13.7	49.5	33.2(31.3,35.1)	4.2(3.4,5.0)
Thailand	2008	2536	2376349	13.6	47.3	37.3(35.4,39.2)	6.1(5.2,7.0)
China	2003	8253	768888	13.7	50.4	21.5(20.6,22.4)	4.2(3.8,4.6)
Overall		59587	16965696	13.8	47.0	32.7(29.0,36.4)	10.3(8.6,12.0)

a. Estimates are based on weighted sample. The outcome for regions and the overall are calculated by meta-analyses. The 95% CI of estimates is enclosed in brackets.

**Fig 2:**
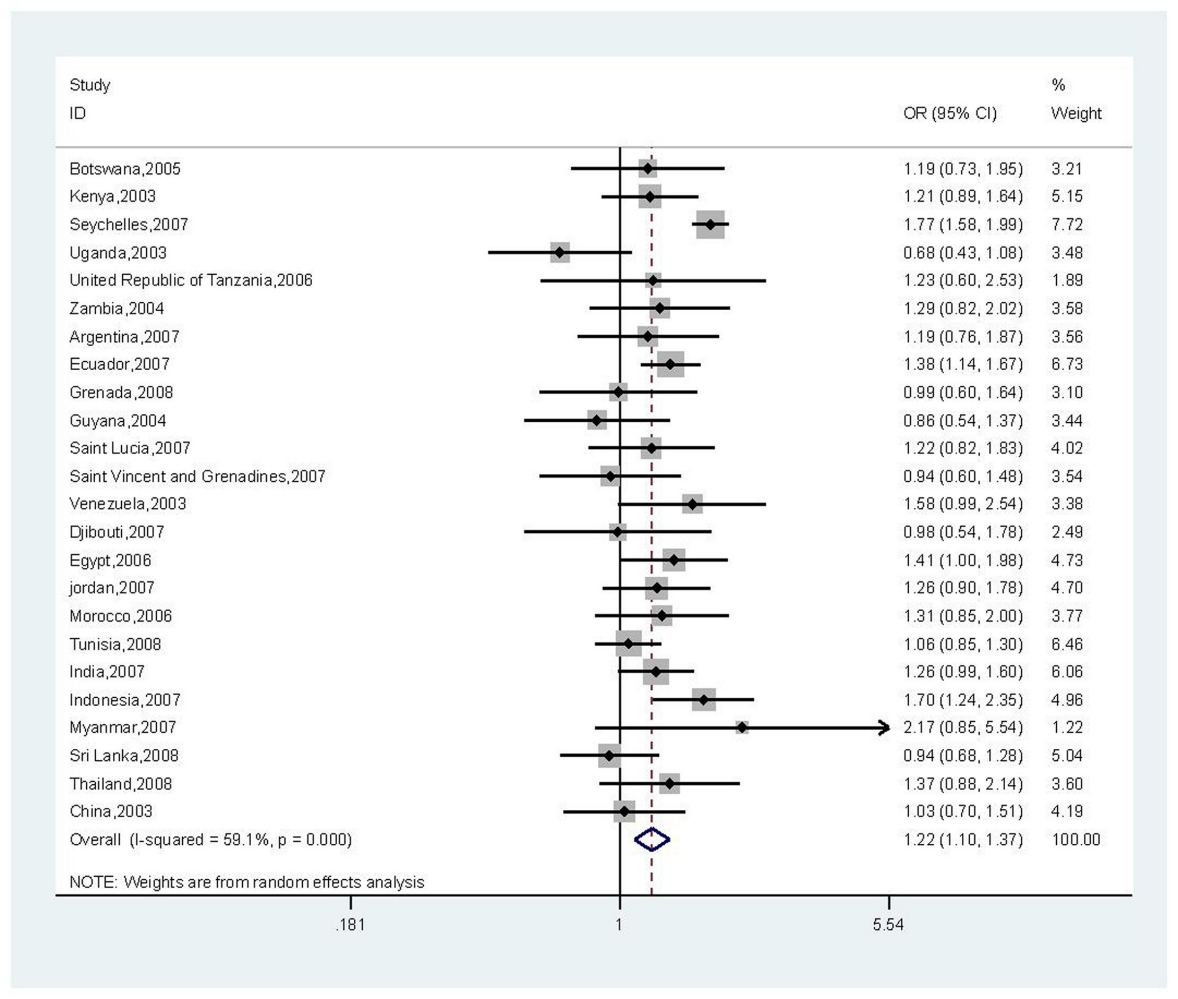
Association between >2 h/d of sedentary behaviour and anxiety symptoms. Country-wise logistic regression models adjusted for age, sex, fruit and vegetable intake and physical activity, being bullied, smoking status(except for Sri Lanka, Venezuela, Egypt and Zambia) and alcohol intake (except for India, Djibouti, Egypt, Jordan, Tunisia and Sri Lanka).

Country-wise analyses found that high sedentary time vs. low sedentary time was significantly associated with a higher risk of anxiety symptoms in 4 countries (Seychelles OR = 1.77, 95% CI = 1.58–1.99; Ecuador, OR = 1.38, 95% CI = 1.14–1.67; Egypt, OR = 1.41, 95% CI = 1.00–1.98; Indonesia, OR = 1.70, 95% CI = 1.24–2.35) out of the 24 LMICs (Fig 2). The pooled OR (95% CI) ground on a meta-analysis was 1.13 (1.07, 1.20).

Subgroup analyses (Table 2) indicate that high sedentary time was significantly associated with an increased risk of anxiety symptoms no matter boys or girls. Nevertheless, there was no significant association of sedentary behaviour with anxiety symptoms in Africa.

**Table 2:** Subgroup analysis for >2 h/d of sedentary behaviour and anxiety symptoms^a^.

	No. of effect sizes	OR (95% CI)	P	I^2^ (%)	P_Heterogeneity_
Sex					
Male	24	1.32(1.22,1.44)	<0.001	0	0.501
Female	24	1.27(1.10,1.47)	0.001	59.4	<0.001
Region					
Africa	6	1.22(0.90,1.66)	0.201	22.2	<0.001
Americas	7	1.21(1.04,1.41)	0.012	11.8	0.340
Eastern Mediterranean	5	1.17(1.01,1.36)	0.031	0	0.586
East and Southeast Asia	6	1.26(1.03,1.55)	0.028	9.31	0.097

a. Results from random-effect meta-analyses based on weighted sample. Country-wise logistic regression models adjusted for age, sex, fruit and vegetable intake, physical activity, being bullied, smoking status(except for Sri Lanka, Venezuela, Egypt and Zambia) and alcohol intake (except for India, Djibouti, Egypt, Jordan, Tunisia and Sri Lanka).

Considering the large sample size, the power values for country-wise analysis are all approaching 1. Nevertheless, the P value of our overall result is <0.001, which means even with decreased α value, the results were still statistically significant.

## 4. Discussion

In this multi-national study of youth, high sedentary time vs low sedentary time was associated with a higher risk of anxiety symptoms after combining effect sizes from 24 LMICs. The positive associations between sedentary behaviour and anxiety symptoms were still significant for both boys and girls when we conducted stratified analyses by sex and region. These results from youth are consistent with previous findings showing a positive association between sedentary behaviour and mental disorders symptoms. Particularly, a study of Canadian adolescents verified an association between increased screen-time and severe symptoms of anxiety [13]. A cross-sectional study found that higher levels of sedentary activity were related to higher anxiety symptoms over time [14]. Additionally, a recent review suggested that sedentary behaviour may be a deleterious factor for anxiety symptoms, independent of physical activity [15]. In contrast to our findings, a cross-sectional study found an inverse association between screen time and anxiety symptoms, indicating that spending more time on screen-based entertainment was connected to a lower risk of anxiety symptoms [16]. Nevertheless, that study focus on a sample of 5-year old children and hence the different target groups may be the reason for the conflicting findings.

The pooled prevalence rate of anxiety symptoms (10.3% vs 6.5%) in the current study was higher than that in a recent meta-analysis [4]. The reasons were as follows: On the one hand, these two researches included youth of different age. The age range of the meta-analysis was 6–18 years, which is even broader than that of our study (12–15 years). On the other hand, evidence has shown that older youngsters are easier to suffer from mental disorders [4]. Besides, a review found that those living in LMICs have a considerably higher prevalence of mental disorders than those living in HICs [17].

There are some potential mechanisms on the association between sedentary behaviour and mental disorders symptoms. For instance, a social withdrawal theory posits that spend too much time on sedentary behaviour may lead to social solitude and withdrawing from interpersonal relationships [15]. Youth may be more vulnerable to physiological responses from the arousal of the central nervous system and spending too much time on-screen viewing may have an adverse effect on sleep patterns [18]. Besides, a previous study suggested those suffering anxiety symptoms tend to spend more time on sedentary behaviour as a way of treating anxiety symptoms [19]. Future research ought to check more earnestly into the underlying mediating mechanisms.

## 5. Strengths and limitations

The strengths of the study include the large sample size (59587) and the multi-national scope. The current study focuses on youth in LMICs. And the relationship between sedentary behaviour and anxiety symptoms among youth was rarely studied. Nonetheless, there are some limitations. First, causal effects cannot be drawn because of the cross-sectional design. High quality longitudinal and intervention studies are needed to confirm our results. Second, the findings of this study may not apply to youth who have no access to school. Third, anxiety symptoms were defined by a single self-report question. The specificity and sensitivity are indistinct compared to the gold standard diagnostic criteria. Lastly, sedentary behaviour was only captured with a self-report measure. Future research ought to utilize objective measures of sedentary behaviour.

## 6. Conclusion

The current study provides multi-national evidence of the dangerous effect of sedentary behaviour on anxiety symptoms among youth in LMICs. This was irrespective of being bullied, smoking status, alcohol intake and physical activity. These findings could contribute to practical intervention strategies for improving the emotional health of youth to enhance the quality of life ultimately. Besides, we offer a significant target for future longitudinal study.

## References

[1] Whiteford HA, Degenhardt L, Rehm J, Baxter AJ, Ferrari AJ, Erskine HE, et al Global burden of disease attributable to mental and substance use disorders: findings from the Global Burden of Disease Study 2010. Lancet. 2013;382(9904):1575–1586. 10.1016/S0140-6736(13)61611-6 23993280

[2] Davies J. How Voting and Consensus Created the Diagnostic and Statistical Manual of Mental Disorders (DSM-III). Anthropol Med. 2017;24(1):32–46. 10.1080/13648470.2016.1226684 27650639

[3] Liu MW, Chen QT, Towne SD Jr, Zhang J, Yu HJ, Tang R, et al Fruit and vegetable intake in relation to depressive and anxiety symptoms among adolescents in 25 low- and middle-income countries. J Affect Disord. 2020;261:172–180. 10.1016/j.jad.2019.10.007 31634676

[4] Polanczyk GV, Salum GA, Sugaya LS, Caye A, Rohde LA. Annual research review: A meta-analysis of the worldwide prevalence of mental disorders in children and adolescents. J Child Psychol Psychiatry. 2015;56(3):345–365. 10.1111/jcpp.12381 25649325

[5] Kessler RC, Berglund P, Demler O, Jin R, Merikangas KR, Walters EE. Lifetime prevalence and age-of-onset distributions of DSM-IV disorders in the National Comorbidity Survey Replication. Arch Gen Psychiatry. 2005;62(6):593–602. 10.1001/archpsyc.62.6.593 15939837

[6] Tremblay MS, LeBlanc AG, Kho ME, Saunders TJ, Larouche R, Colley RC, et al. Systematic review of sedentary behaviour and health indicators in school-aged children and youth. Int J Behav Nutr Phys Act. 2011;8:98 10.1186/1479-5868-8-98 21936895 PMC3186735

[7] Tremblay MS, Aubert S, Barnes JD, Saunders DJ, Carson V, Latimer-Cheung AE, et al Sedentary Behavior Research Network (SBRN)—Terminology Consensus Project process and outcome. Int J Behav Nutr Phys Act. 2017;14(1):75 10.1186/s12966-017-0525-8 28599680 PMC5466781

[8] Guthold R, Cowan MJ, Autenrieth CS, Kann L, Riley LM. Physical activity and sedentary behavior among schoolchildren: a 34-country comparison. J Pediatr. 2010;157(1):43–49.e1. 10.1016/j.jpeds.2010.01.019 20304415

[9] Zink J, Belcher BR, Imm K, Leventhal AM. The relationship between screen-based sedentary behaviors and symptoms of depression and anxiety in youth: a systematic review of moderating variables. BMC Public Health. 2020;20(1):472 10.1186/s12889-020-08572-1 32272906 PMC7147040

[10] McVeigh J, Smith A, Howie E, Straker L. Trajectories of Television Watching from Childhood to Early Adulthood and Their Association with Body Composition and Mental Health Outcomes in Young Adults. PLoS One. 2016;11(4):e0152879 10.1371/journal.pone.0152879 27097324 PMC4838324

[11] Barbosa Filho VC, Minatto G, Mota J, Silva KS, de Campos W, Lopes Ada S. Promoting physical activity for children and adolescents in low- and middle-income countries: An umbrella systematic review: A review on promoting physical activity in LMIC. Prev Med. 2016;88:115–126. 10.1016/j.ypmed.2016.03.025 27068650

[12] Rodriguez-Ayllon M, Cadenas-Sánchez C, Estévez-López F, Muñoz NE, Mora-Gonzalez J, Migueles JH, et al Role of Physical Activity and Sedentary Behavior in the Mental Health of Preschoolers, Children and Adolescents: A Systematic Review and Meta-Analysis. Sports Med. 2019;49(9):1383–1410. 10.1007/s40279-019-01099-5 30993594

[13] Maras D, Flament MF, Murray M, Buchholz A, Henderson KA, Obeid N, et al Screen time is associated with depression and anxiety in Canadian youth. Prev Med. 2015;73:133–138. 10.1016/j.ypmed.2015.01.029 25657166

[14] Cao H, Qian Q, Weng T, Yuan C, Sun Y, Wang H, et al Screen time, physical activity and mental health among urban adolescents in China. Prev Med. 2011;53(4–5):316–320. 10.1016/j.ypmed.2011.09.002 21933680

[15] Teychenne M, Costigan SA, Parker K. The association between sedentary behaviour and risk of anxiety: a systematic review. BMC Public Health. 2015;15:513 10.1186/s12889-015-1843-x 26088005 PMC4474345

[16] Griffiths LJ, Dowda M, Dezateux C, Pate R. Associations between sport and screen-entertainment with mental health problems in 5-year-old children. Int J Behav Nutr Phys Act. 2010;7:30 10.1186/1479-5868-7-30 20409310 PMC2867988

[17] Yatham S, Sivathasan S, Yoon R, da Silva TL, Ravindran AV. Depression, anxiety, and post-traumatic stress disorder among youth in low and middle income countries: A review of prevalence and treatment interventions. Asian J Psychiatr. 2018;38:78–91. 10.1016/j.ajp.2017.10.029 29117922

[18] Vancampfort D, Stubbs B, Firth J, Van Damme T, Koyanagi A. Sedentary behavior and depressive symptoms among 67,077 adolescents aged 12–15 years from 30 low- and middle-income countries. Int J Behav Nutr Phys Act. 2018;15(1):73 10.1186/s12966-018-0708-y 30089487 PMC6083627

[19] Sabiston CM, Sedgwick WA, Crocker PRE, Kowalski KC, Mack DE. Social physique anxiety in adolescence: an exploration of influences, coping strategies, and health behaviors. J Adolesc Res. 2007;22(1):78–101.

